# Supplementary material for: Chemical Variability of Peel and Leaf Essential Oils in the Citrus Subgenus Papeda (Swingle) and Few Relatives
Source: Plants (Basel). 2021 May 31;10(6):1117. doi: 10.3390/plants10061117 (PMC8227882; doi:10.3390/plants10061117)
Supplement: Supplementary file 1 [file plants-10-01117-s001.zip › plants-1238549-supplementary.pdf]

plants

Supplementary Materials

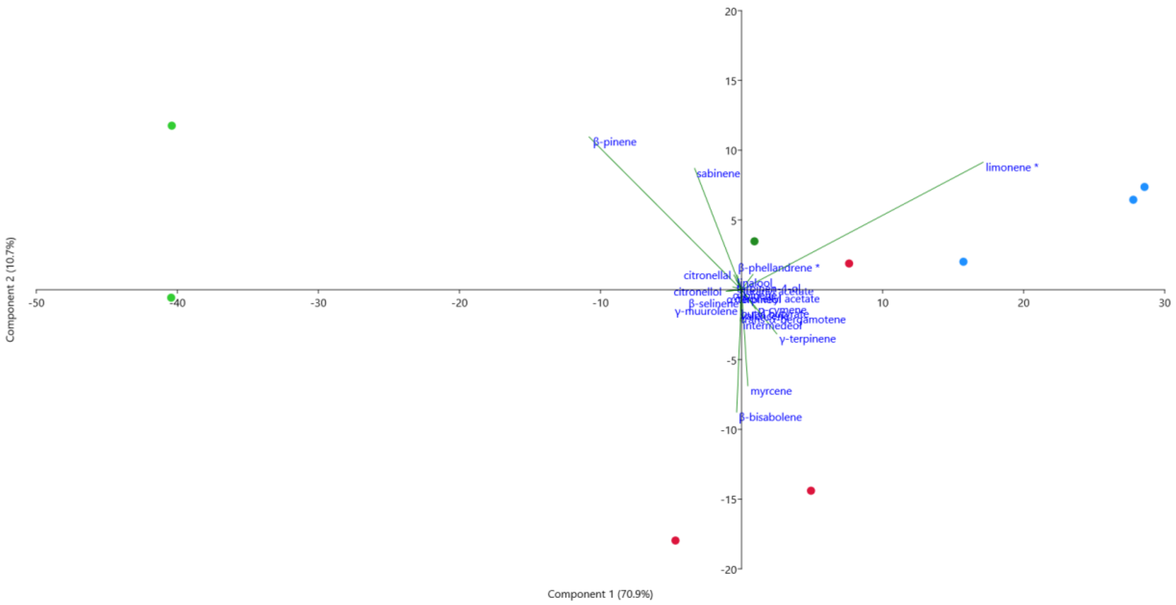

Figure S1. Variable contributions in PCA of peel oils. Green: hys: C. hystrix, mic: C. micrantha, mapt: C. macroptera; Red: lat: C. latipes, ich: C. ichangensis; Blue: wil: C. wilsonii, jun: C. junos, maph: C. macrophylla.

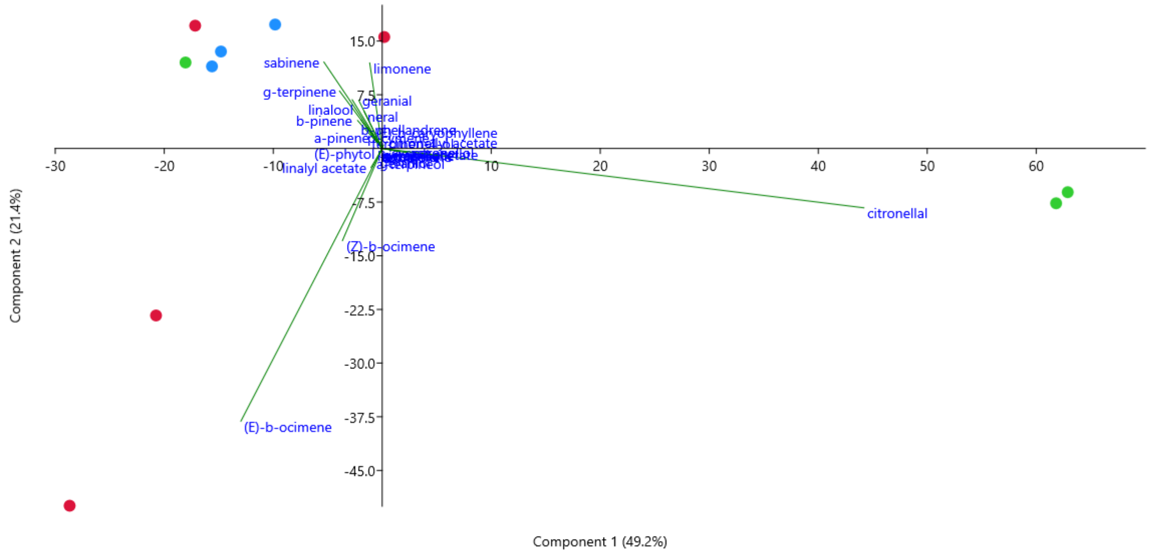

Figure S2. Variable contributions in PCA of leaf oils. Green: hys: C. hystrix, mic: C. micrantha, mapt: C. macroptera; Red: lat: C. latipes, ich: C. ichangensis; Blue: wil: C. wilsonii, jun: C. junos, maph: C. macrophylla.
